# Assessing Genotoxicity of Ten Different Engineered Nanomaterials by the Novel Semi-Automated FADU Assay and the Alkaline Comet Assay

**DOI:** 10.3390/nano12020220

**Published:** 2022-01-10

**Authors:** Sarah May, Cordula Hirsch, Alexandra Rippl, Alexander Bürkle, Peter Wick

**Affiliations:** 1 Particles-Biology Interactions Lab, Swiss Federal Laboratories for Materials Science and Technology (EMPA), Lerchenfeldstrasse 5, 9014 St. Gallen, Switzerland; sf.may@hotmail.de (S.M.); cordula.hirsch@empa.ch (C.H.); alexandra.rippl@empa.ch (A.R.); 2 Molecular Toxicology Group, University of Konstanz, Universitätsstrasse 10, 78464 Konstanz, Germany; alexander.buerkle@uni-konstanz.de

**Keywords:** comet assay, FADU assay, engineered nanomaterials, DNA strand breaks, genotoxicity, ENM interference

## Abstract

Increased engineered nanomaterial (ENM) production and incorporation in consumer and biomedical products has raised concerns about the potential adverse effects. The DNA damaging capacity is of particular importance since damaged genetic material can lead to carcinogenesis. Consequently, reliable and robust in vitro studies assessing ENM genotoxicity are of great value. We utilized two complementary assays based on different measurement principles: (1) comet assay and (2) FADU (fluorimetric detection of alkaline DNA unwinding) assay. Assessing cell viability ruled out false-positive results due to DNA fragmentation during cell death. Potential structure–activity relationships of 10 ENMs were investigated: three silica nanoparticles (SiO_2_-NP) with varying degrees of porosity, titanium dioxide (TiO_2_-NP), polystyrene (PS-NP), zinc oxide (ZnO-NP), gold (Au-NP), graphene oxide (GO) and two multi-walled carbon nanotubes (MWNT). SiO_2_-NPs, TiO_2_-NP and GO were neither cytotoxic nor genotoxic to Jurkat E6-I cells. Quantitative interference corrections derived from GO results can make the FADU assay a promising screening tool for a variety of ENMs. MWNT merely induced cytotoxicity, while dose- and time-dependent cytotoxicity of PS-NP was accompanied by DNA fragmentation. Hence, PS-NP served to benchmark threshold levels of cytotoxicity at which DNA fragmentation was expected. Considering all controls revealed the true genotoxicity for Au-NP and ZnO-NP at early time points.

## 1. Introduction

Due to their novel and unique physico-chemical properties, engineered nanomaterials (ENMs) are remarkable for their rapid technological development accompanied by increasing production in the last two decades. Because of their small size, particulate shape, increased surface area and surface reactivity, some of these new nanosized materials might cause different biological effects compared to the bulk, composite or ionic form. Nevertheless, many ENMs find application in diverse areas, such as energy, information technology, electronics, cosmetics and healthcare, e.g., as diagnostics or therapeutics (for an overview see [1] and references therein). Despite the undeniable benefits of ENMs for many applications, there is a growing concern that these novel properties may cause harm to human beings and the environment [1,2,3,4,5,6]. The conflictive literature on ENM toxicity impedes their evaluation by regulatory bodies and hinders the efficient transfer of nano-based applications into the clinic. For these reasons, the toxicological impact of ENMs needs to be thoroughly investigated by in vitro and in vivo studies. In particular, the evaluation of ENM genotoxicity in human cells is of great importance since genotoxic insults independent of their origin are clearly linked to adverse health effects, most notably to cancer development [7]. Over the past decades, the literature on ENM-induced genotoxicity has grown but the results remain inconsistent, inconclusive or even contradictory. Numerous reviews, surveying the literature suggests diverse factors influencing the obtained results [5,8,9,10,11,12]. Accordingly, contradictory findings of different studies can be explained by the variability in physico-chemical characteristics of ENMs, for instance in size, shape, structure and coating and the type of biological model systems used. Furthermore, the unique characteristics of ENMs increase their likelihood of interference with analytical methods, detection systems and assay components, which may lead to data artefacts and inconsistent results of genotoxicological assays [13,14,15]. In some cases, the variability of experimental results between different laboratories might originate from the diversity of methods for handling ENM and the differences in dispersion protocols [16]. The most important factor, however, is the lack of standardized assay conditions and protocols [17]. Therefore, extensive investigations on ENM genotoxicity with appropriate assay systems in human cells are still required and significant [5,18].

Genotoxicity is a complex research field comprising not only the detection of specific DNA lesions (DNA strand breaks, alkali-labile sites [ALS] or chromosomal aberrations to name only a few) but also including modes of DNA repair. Here, we focus particularly on the detection of DNA strand breaks as one well-known type of DNA damage. In this context, two assays shall be highlighted: the comet (or single-cell gel electrophoresis) assay which is one of the most used assays for the detection of DNA strand breaks and the FADU (fluorimetric detection of alkaline DNA unwinding) assay as a new emerging semi-automated technology [19,20]. The comet assay was originally developed in 1984 by Ostling and Johanson and further modified in 1988 by Singh and colleagues [21,22] and is commonly used to determine the induction of DNA breakage by genotoxic agents and ionizing radiation at the level of individual cells. The assay principle is relatively straightforward. An OECD guideline (TG 489) [23] for an in vivo version exists and detailed publications on technical aspects for the in vivo and in vitro assay are available [24]. Therefore, this method has become the most frequently used method for in vitro ENM genotoxicity [25]. However, the assay itself and the evaluation of the results is quite tedious and time-consuming. Furthermore, several interlaboratory comparison studies have demonstrated the poor reproducibility of this assay [26,27,28,29]. The FADU assay was first described by Birnboim and Jevcak in 1981 [30] and is based on the progressive unwinding of DNA under the controlled conditions of time, temperature and alkaline pH. Besides replication forks and chromosome ends, DNA strand breaks are the origin of the unwinding process. For the evaluation of DNA damage, a fluorescent dye intercalating preferentially into double-stranded DNA (e.g., SybrGreen^®^) is used. A decrease in SybrGreen^®^ fluorescence intensity in the cell lysates indicates an increase in progressive DNA unwinding and, thus, represents a greater number of DNA strand breaks. The applicability of this method has been demonstrated for the detection of mutagen-induced DNA strand breaks [20,31], the repair of UV-induced DNA strand breaks [32], environmental genotoxic effects [33] and ZnO nanoparticles [34]. The novel semi-automated version of this assay enables the fast assessment of DNA breakage in a 96-well-plate format and the integration of different control samples for ENM interference detection. Very recent research has extended its field of application to the detection of oxidative and methylation-induced DNA damage [19].

The goal of this case study was to assess the potential of a broad range of nanomaterials to induce DNA strand breaks in vitro in order to provide the first data set on potential structure–activity relationships (SAR). Physico-chemical properties of interest included differences in ENM chemistry, size, shape and porosity. Therefore, the following set of ENM was chosen: two metal oxide nanoparticles, non-soluble and soluble (TiO_2_-NP and ZnO-NP) that are produced in high quantities. Three carbon-based materials, i.e., graphene oxide (GO) as a novel and highly interesting 2-D material, and two different multi-walled carbon nanotubes (MWNTs) as well-known benchmark materials. A medically relevant gold NP (Au-NP I) that had been shown to induce DNA strand breaks in a previous study [35]. Amine-modified polystyrene NPs (PS-NP) as a known cytotoxic material as well as three types of silica NPs (SiO_2_-NPs) of low, middle and high porosity.

Upon ENM exposure, their interaction with cells of the immune system was very likely [36]. Therefore, we chose the T-lymphocytic cell line Jurkat E6-I as one potential immune system candidate to make contact with ENMs early on. The sublethal working concentrations of all ENM were determined using the MTT viability assay. DNA fragmentation occurring during cell death is the main cause of false-positive genotoxicity results. Therefore, ruling out cytotoxicity was indispensable for a reliable interpretation of gentoxicity data. Furthermore, two complementary assays to assess DNA strand breaks that are based on different read-out systems were used, namely the well-established comet assay and the semi-automated FADU assay. Thus, assay intrinsic errors such as ENM interferences could be detected and potentially even avoided which would increase the reliability and robustness of the acquired data sets [37]. Consolidating results from both methods finally facilitated a better fundamental understanding of the DNA-damaging potential of ENM.

## 2. Materials and Methods

**Cell Culture:** The Jurkat E6-I lymphoblastoid cell line (ATCC: TIB-152) was purchased from the American Type Culture Collection (Manassas, VA, USA). Cells were maintained in a complete medium (CM) consisting of RPMI (Roswell Park Memorial Institute) 1640 medium (Gibco) supplemented with 10% fetal bovine serum (FBS, Invitrogen) and 1% penicillin/streptomycin/neomycin (Gibco) at standard growth conditions of 37 °C and 5% CO_2_ in a humidified atmosphere. Jurkat E6-I cells were cultured in suspension and cell titers were not allowed to exceed 3 × 10^6^ cells/mL.

**ENM handling and Dilutions:** ENMs, delivered as powder, were prepared as 1 mg/mL stock suspensions either in ddH_2_O or 160 ppm Pluronic F-127 (Sigma), as summarized in Table 1. ZnO, TiO_2_, MWNT A and MWNT C suspensions were ultrasonicated in an ultrasound bath (Bandelin electronic, Berlin, Germany) for 10 min. GO suspension was ultrasonicated for 2 min. ENM stock suspensions were serially pre-diluted in the respective solvent. The same volume from different pre-dilutions and solvents was then used to prepare the final concentrations in CM. All suspensions were prepared and diluted directly before application to cells. To reduce formation of aggregates, tubes containing the solvent were placed on a continuously shaking Vortex^®^ (Witeg, Wertheim am Main, Germany) as described by Zook and co-workers in 2011 [38]. The stock suspensions or respective sub-dilutions were added to the shaking solvent in a drop-wise manner. The resulting suspension remained on the Vortex^®^ for an additional 3 s. Directly before application to cells, all final suspensions were vortexed again.

**MTT Assay:** The cell viability of Jurkat E6-I cells was determined by measuring the reduction of water-soluble MTT (3-(4,5-dimethylthiazol-2-yl)-2,5-diphenyltetrazolium bromide, Sigma) to water-insoluble formazan by metabolically active cells. Briefly, 7 × 10^4^ Jurkat E6-I cells were seeded in 96-well plates in a volume of 50 µL directly before treatment with 50 µL of double-concentrated ENM suspensions. ZnO, TiO_2_, PS and GO stock suspensions were serially diluted 1:2 as described above and reached final concentrations in a medium of 100, 50, 25, 12.5, 6.25, 3.125, 1.56, 0 μg/mL. The MWNTs suspensions were serially diluted in Pluronic F-127 and reached final assay concentrations in a medium of 50, 25, 12.5, 6.25, 3.125, 1.56, 0.78, 0 μg/mL. “0 µg/mL” samples received the corresponding amount of the respective solvent. Following incubation for 30 min or 21 h, 10 µL of MTT solution (5 mg/mL in PBS) were added and cells were incubated for 3 h under standard growth conditions. Sodium dodecyl sulfate (SDS, Sigma, 200 µM) served as positive control. After incubation, 100 µL of solubilizing solution (10% SDS in 0.01 M HCl) was added and the samples were incubated at 37 °C overnight. Absorbance was measured at 590 nm and 750 nm as references (Mithras2 LB943, Berthold Technologies, Bad Wildbad, Germany). Wells without cells were used as blanks and subtracted from the corresponding sample values. The mean and the corresponding standard deviation of at least three independent experiments with six technical replicates each are shown.

**Comet Assay:** The alkaline comet assay was performed as previously described by Singh and co-workers in 1988 [22] with the following modifications. Jurkat E6-I cells were seeded in 6-well plates at a density of 5 × 10^5^ cells per well in 1.25 mL CM directly before ENM application. For treatment, a volume of 1.25 mL double-concentrated ENM suspension was added to reach the following final concentrations for ZnO, TiO_2_, amine-modified PS, GO, SiO_2_ and Au: 100, 6.25, 3.125, 1.56 μg/mL and 50, 3.125, 1.56, 0.78 μg/mL for MWNTs. The application of 20 mM EMS (Sigma) 30 min before the end of the incubation time served as the positive control. Following 3 h or 24 h of incubation, cells were collected and centrifuged for 6 min at 125× *g*. The pellets obtained were resuspended in 300 µL CM. All subsequent steps were identical to the protocol described by May and co-workers in 2018 [35]. If not otherwise stated, samples were blinded and 100 randomly chosen comets per sample were analyzed for each experiment. Tail intensities in percentages (=tail intensity (%)) are expressed as the mean of at least three independent experiments and their corresponding standard deviations.

**FADU Assay:** The automated FADU assay was performed according to the protocol published with minor modifications [20,34]. Directly prior to experimentation 3.6 × 10^4^ Jurkat E6-I cells were seeded into deep-well 96-well plates (Greiner) in a volume of 80 µL CM. Five-times-concentrated ENM pre-dilutions were prepared in CM. 20 µL of these suspensions were added to the 80 µL of cells to reach final ENM concentrations of 100, 50, 25, 12.5, 6.25, 3.125, 1.56, 0 μg/mL in a total volume of 100 µL CM. The final concentrations of MWNTs differed from those of all other ENM and equaled 50, 25, 12.5, 6.25, 3.125, 1.56, 0.78, 0 μg/mL. Etoposide (20 µM, 30 min) served as the chemical positive control. Untreated samples received CM only. After 3 h and 24 h, DNA strand break analysis using the AUREA gTOXXs Anlayzer (3T analytik; www.aurea.solutions (accessed on 27 October 2021) was carried out. Fluorescence measurements were performed using a multi-well plate reader (Mithras^2^, Berthold Technologies) with an excitation wavelength of 492 nm and an emission of 530 nm. The overall fluorescence intensity of the *p*-values was expressed as the percentage of the fluorescence of the control cells, i.e., cells that were not been exposed to ENM. A decrease in the fluorescence intensity indicated an increase in DNA strand breaks. Two different conditions were applied for each sample, one in which the DNA was not unwound and, therefore, represents the total amount of double-stranded DNA (T-value), and one in which the DNA was unwound by the addition of unwinding solution prior to the neutralization solution (*p*-value). Only for the assessment of ENM-induced interference were the T-values and *p*-values used. To take the potential ENM-derived influence (fluorescence quenching or enhancement) to the resulting *p*-values into account, each *p*-value was expressed as the percentage of its respective T-value before being normalized to the solvent control as described above. Results processed like this are declared as “corrected”. In all cases, data shown represent the mean and corresponding standard deviation of at least three independent experiments with four technical replicates each. 

**Statistical Analysis:** Microsoft Excel (2016) was used for figures and statistical calculations. Statistical differences were assessed by the Student’s *t*-test. *p* values ≤ 0.05 were considered as statistically significant. For FADU and viability (MTT) assessments, an additional criterion for “biological significance” was used, i.e., only reductions in cell viability and intact DNA below 80% were considered biologically relevant. Since variability in the comet assay is known to be high [39], only 2-fold differences compared to the untreated control were considered relevant.

## 3. Results

### 3.1. ENM Characterization

For this study, a panel of ten different ENMs was investigated regarding their ability to induce DNA damage in Jurkat E6-I cells. The characterization of these materials was published earlier and is summarized in Table 2 and Table 3.

### 3.2. ENM Influence on Cell Viability and DNA Damage

The cell viability of Jurkat E6-I cells was investigated after 3 and 24 h of treatment with different ENMs by using the MTT assay. Upon viability assessment, and for the ease of reading, data are grouped throughout the manuscript according to ENM cytotoxicity. Firstly, results from non-cytotoxic ENMs, including SiO_2_-160, MS-SiO_2_-140, MSHT-SiO_2_-300, TiO_2_-NP and GO are presented, followed by results from the cytotoxic panel consisting of Au-NP I, MWNT A, MWNT C, ZnO-NP and PS-NP. 

**Figure 1:**
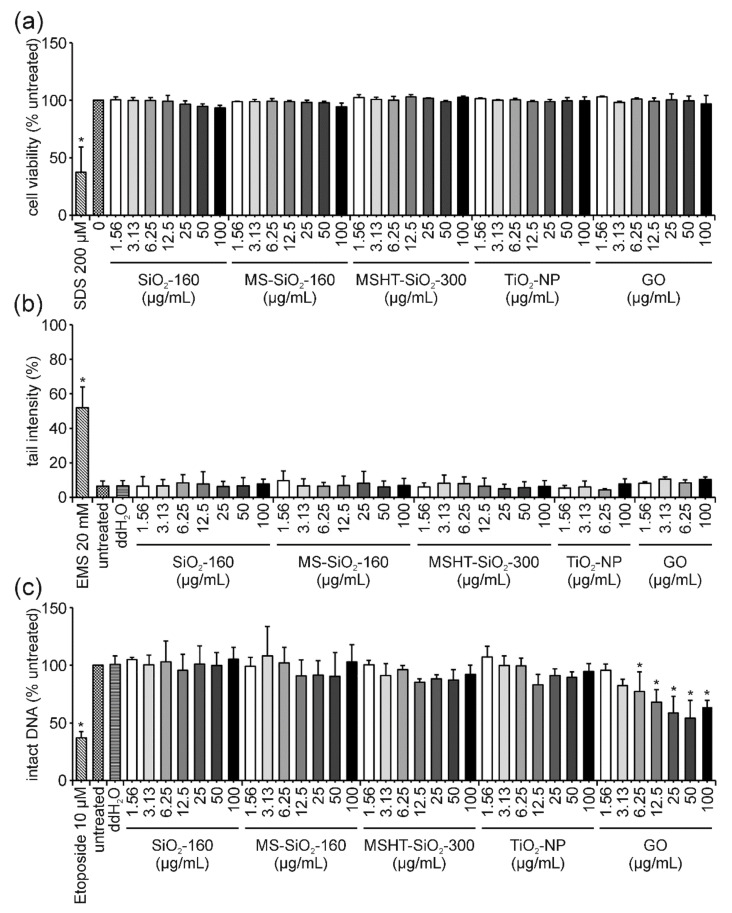
**Influence of SiO_2_-160, MS-SiO_2_-160, MSHT-SiO_2_-300, TiO_2_ and GO on Jurkat E6-I cell viability and DNA damage induction after 3 h of incubation**. Following the incubation of Jurkat E6-I cells with different concentrations of SiO_2_-160, MS-SiO_2_-160, MSHT-SiO_2_-300, TiO_2_-NP and GO for 3 h, cell viability was determined by MTT assay (**a**). As a positive control, cells were incubated with 200 µM sodium dodecylsulfate (SDS, 3 h). DNA damage expressed as tail intensity percentage was assessed by alkaline comet assay (**b**). Ethyl methanesulfonate (EMS, 30 min) served as the positive control. The FADU assay was performed as a second method for genotoxicity assessment (**c**). Treatment with etoposide (30 min) served as the positive control. Results represent the mean and corresponding standard deviations from at least three independent experiments. (* *p* ≤ 0.05).

No decrease in cell viability could be observed after 3 h of incubation with the first group of ENMs (Figure 1a). The alkaline comet assay revealed the absence of DNA damage after 3 h of exposure to the same set of ENMs (Figure 1b). Similarly, in the FADU assay, no significant reduction of intact DNA was observed for the three types of silica particles (SiO_2_-160, MS-SiO_2_-160 and MSHT-SiO_2_-300) and TiO_2_-NP (Figure 1c). A significantly strong dose-dependent decrease in the percentage of intact DNA was detected for GO, starting at a concentration of 3.13 µg/mL; however, for the highest concentration of 100 µg/mL, a slight increase was observed (Figure 1c).

**Figure 2:**
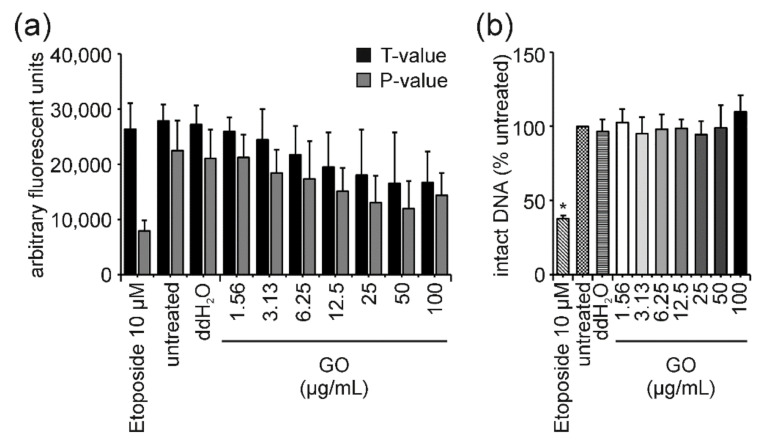
**GO-induced interference and interference correction in the FADU assay after 3 h of incubation in Jurkat E6-I cells**. Following 3 h exposure of Jurkat E6-I cells to GO, the FADU assay was performed and revealed a dose-dependent decrease in fluorescence of T- and *p*-values (**a**). After correction of the observed interference, no reduction in intact DNA was observed for any concentration of GO (**b**). Only incubation with 10 µM etoposide for 30 min, which served as the positive control, induced genotoxic effects. Data shown represent the mean of three independent experiments and the corresponding standard deviation. (* *p* ≤ 0.05).

In the case of GO-treated cells, the interference correction of FADU results proved to be necessary because this material quenched the fluorescence signal. This could be observed by a dose-dependent reduction in T-values, which is a strong indication for fluorescence quenching (Figure 2a). T-values represent the total amount of DNA, which is obtained by preventing DNA unwinding through the neutralization of an alkaline unwinding buffer. Therefore, these T-values should have stayed equally high for all samples, even genotoxic ones, as double-stranded DNA remains unwound. Only *p*-values, where DNA unwinding is allowed to take place, decrease following treatment with genotoxic stimuli. Consequently, a reduction in T-values indicates either ENM interference in the form of fluorescence quenching or variations in cell density per well. Since cell viability was not influenced by GO treatment, fluorescence quenching seemed to be the reason for the observed decrease in T-values. Following interference correction of the results obtained for GO, according to calculations summarized in materials and methods, no reduction in intact DNA could be detected anymore (Figure 2b).

**Figure 3:**
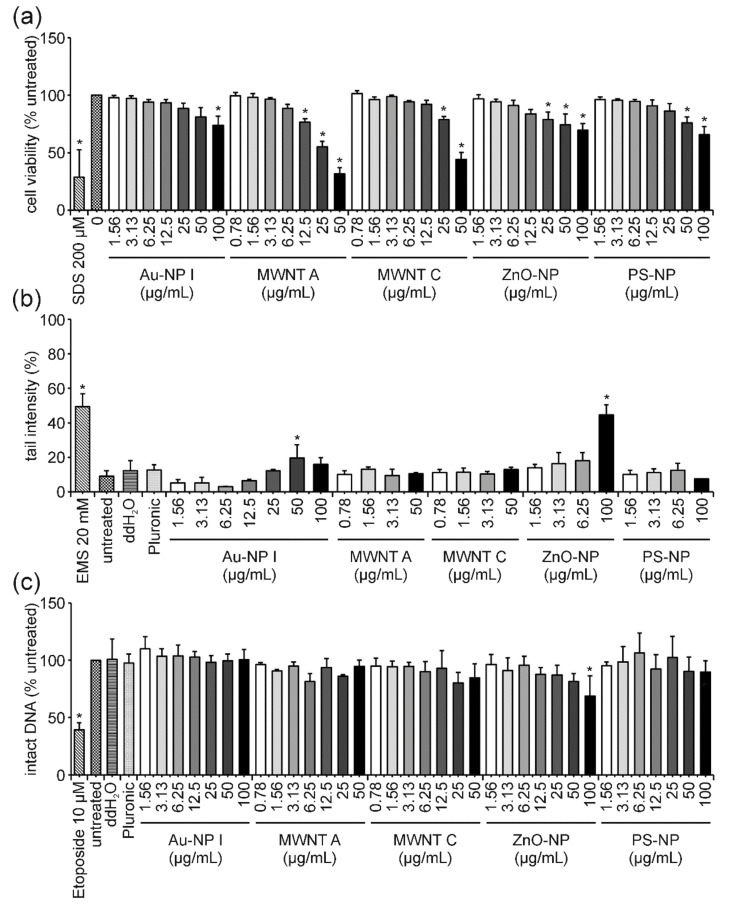
**Influence of Au-NP I, MWNT A, MWNT C, ZnO-NP and PS-NP on Jurkat E6-I cell viability and DNA damage induction after 3 h of incubation**. Following incubation of Jurkat E6-I cells with different concentrations of Au-NP I, MWNT A, MWNT C, ZnO-NP and PS-NP for 3 h, cell viability was determined by MTT assay (**a**). As a positive control, cells were incubated with 200 µM SDS (3 h). DNA damage expressed as tail intensity percentage was assessed by alkaline comet assay (**b**). EMS (30 min) served as the positive control. The FADU assay was performed as a second method for genotoxicity assessment (**c**). Treatment with etoposide (30 min) served as the positive control. Results represent the mean and corresponding standard deviations from at least three independent experiments. (* *p* ≤ 0.05).

After 3 h exposure to Au-NP I, a slight dose-dependent decrease in cell viability was observed (Figure 3a). The highest concentration of 100 µg/mL led to a moderate and statistically significant reduction in cell viability to 74%. A stronger decrease in cell viability was observed for both MWNTs. Cytotoxic effects started at concentrations of 12.5 and 25 µg/mL and declined to 32 and 44% for 50 µg/mL MWNT A and MWNT C, respectively. Additionally, ZnO-NP and PS-NP treatment influenced cell viability negatively and both materials caused similar dose-dependent effects. The highest concentration of 100 µg/mL reduced cell viability to 70 and 66% for ZnO-NP and PS-NP, respectively. Subsequently, the DNA-damaging potential of these ENMs was addressed in the comet assay. In comparison to the untreated and vehicle controls (ddH_2_O for Au-NP I, ZnO-NP and PS-NP; Pluronic F-127 for MWNT A and MWNT C) an increase in DNA damage expressed as tail intensity percentage was observed for Au and in particular for ZnO-NP (Figure 3b). Au treatment resulted in 20 and 16% tail intensity for 50 and 100 µg/mL, respectively. The highest concentration of ZnO-NP reached values of approximately 45%. A very slight and insignificant dose-dependent increase was observed for sublethal concentrations of ZnO-NP (1.56, 3.13 and 6.25 µg/mL) with tail intensities of 14, 16 and 18%, respectively. DNA damage induction was not detected for the two MWNTs or for PS-NP. Results of the FADU assay revealed no reduction in intact DNA for Au-NP I, PS-NP, MWNT A and C (Figure 3c). The strongest, yet still weak dose-dependent effect, was observed for ZnO-NP where the highest concentration resulted in 69% of intact DNA compared to the untreated control (Figure 3c).

**Figure 4:**
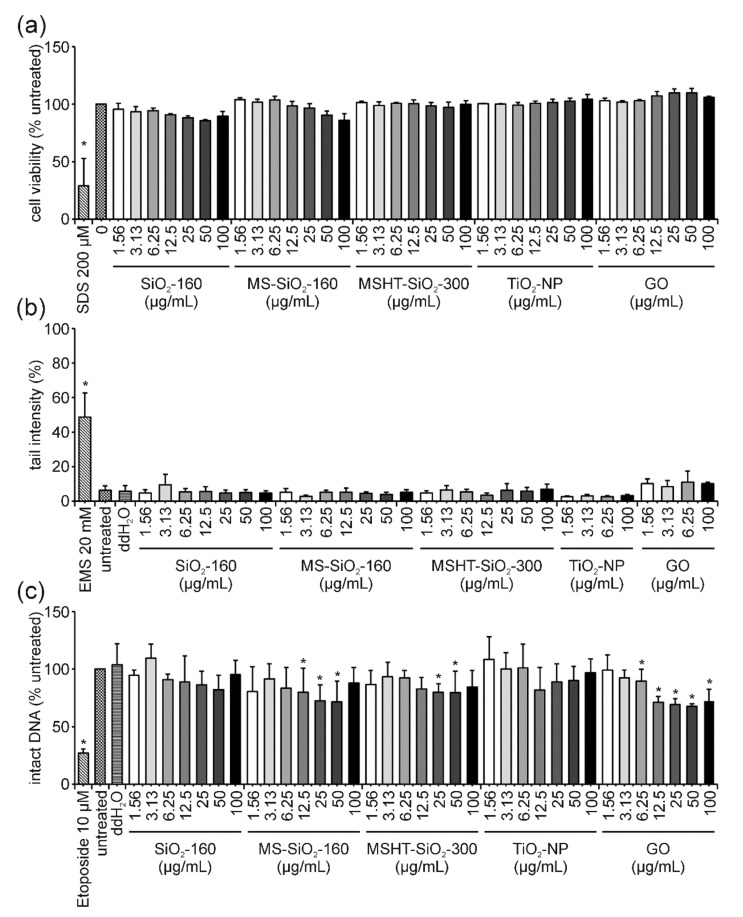
**Influence of SiO_2_-160, MS-SiO_2_-160, MSHT-SiO_2_-300, TiO_2_-NP and GO on Jurkat E6-I cell viability and DNA damage induction after 24 h of incubation**. Following incubation of Jurkat E6-I cells with different concentrations of SiO_2_-160, MS-SiO_2_-160, MSHT-SiO_2_-300, TiO_2_-NP and GO for 24 h, cell viability was determined by MTT assay (**a**). As a positive control, cells were incubated with 200 µM SDS (24 h). DNA damage expressed as tail intensity percentage was assessed by alkaline comet assay (**b**). EMS (30 min) served as the positive control. The FADU assay was performed as a second method for genotoxicity assessment (**c**). Treatment with etoposide (30 min) served as the positive control. Results represent the mean and corresponding standard deviations from at least three independent experiments. (* *p* ≤ 0.05).

The same set of experiments was conducted after 24 h of incubation with all ENMs under investigation. Regarding cell viability, no significant reduction was observed for SiO_2_-160, MS-SiO_2_-140, MSHT-SiO_2_-300, TiO_2_ and GO after 24 h (Figure 4a). Using the comet assay, no formation of DNA damage could be detected for any of these ENMs after 24 h of incubation (Figure 4b). Results of the FADU assay confirmed this observation for SiO_2_-160, MSHT-SiO_2_-300 as well as TiO_2_-NPs (Figure 4c). A minor, yet significant effect could only be detected for treatment with 12.5, 25 and 50 µg/mL MS-SiO_2_-160 resulting in 79.9% and 72% intact DNA, respectively. At the highest concentration of 100 µg/mL, the level of intact DNA increased again to 88%. Similarly, MSHT-SiO_2_-300-treatment resulted in 79% intact DNA at concentrations of 25 and 50 µg/mL, respectively, increasing again to 85% at 100 µg/mL. Considering the rather significant variability (indicated as standard deviation in Figure 4c), results have to be interpreted with caution. Since no increase in tail intensity could be detected with the more sensitive comet assay, the massive DNA-damaging potential of the silica particles could be excluded. However, interference reactions in the FADU assay should be addressed in more detail in the future. Comparable to the 3 h time point, GO-treatment for 24 h again displayed the most pronounced effects in the FADU assay. However, as shown for the 3 h time point, the dose-dependent decrease was, once more, not only detectable for the actual samples (*p*-values) but also for the interference control samples (T-values) (Supplementary Figure S1a). Therefore, interference correction of GO results was performed, which consequently eliminated the initially observed reduction in intact DNA (Supplementary Figure S1b). For all other analyzed ENMs, T-values revealed no decrease in fluorescence, indicating the absence of quenching effects. Hence, interference correction was not essential for those samples.

**Figure 5:**
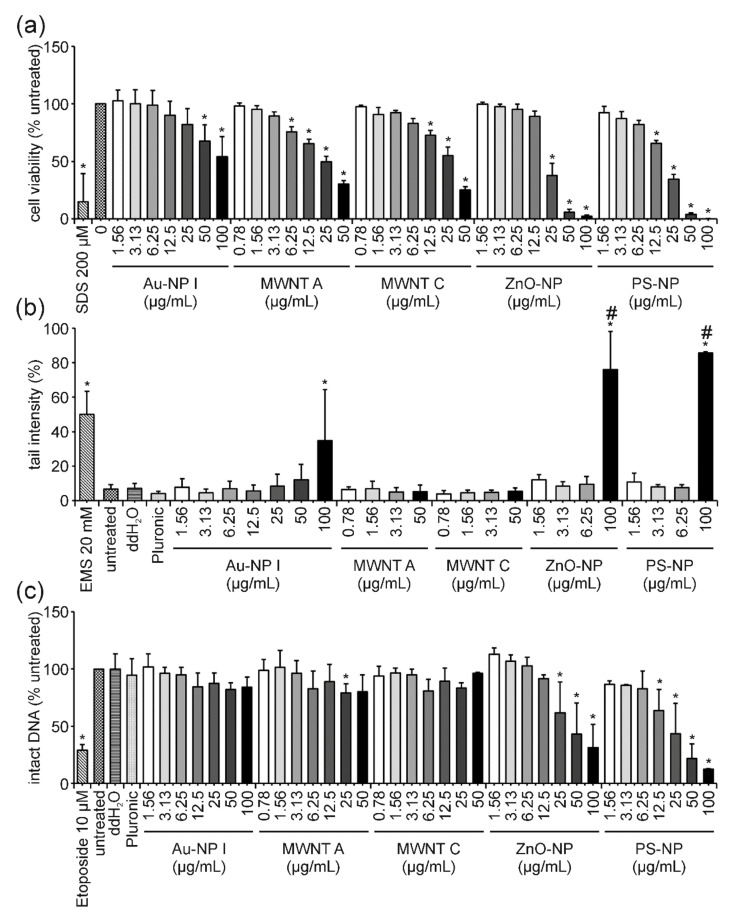
**Influence of Au-NP I, MWNT A, MWNT C, ZnO-NP and PS-NP on Jurkat E6-I cell viability and DNA damage induction after 24 h of incubation**. Following incubation of Jurkat E6-I cells with different concentrations of Au-NP I, MWNT A, MWNT C, ZnO-NP and PS-NP for 24 h, cell viability was determined by MTT assay (**a**). As a positive control, cells were incubated with 200 µM SDS (24 h). DNA damage expressed as tail intensity percentage was assessed by alkaline comet assay (**b**). EMS (30 min) served as the positive control. The FADU assay was performed as a second method for genotoxicity assessment (**c**). Treatment with etoposide (30 min) served as the positive control. Results represent the mean and corresponding standard deviations from three independent experiments. # Only a reduced number of comets (i.e., less than 100) could be counted per experiment in these samples. (* *p* ≤ 0.05).

Significant dose-dependent effects on cell viability were detected after 24 h of exposure to Au-NP I, MWNT A, MWNT C, ZnO-NP and PS-NP (Figure 5a). The reduction in cell viability was more pronounced for all analyzed ENMs after 24 h in comparison to the 3 h measurement. The weakest effect was observed for Au-NP I, while similar results were obtained for both MWNTs. The strongest effects were observed for ZnO-NP and PS-NP. The highest concentration of Au-NP I, MWNT A, MWNT C and ZnO-NP resulted in values of 54, 30, 25 and 2% cell viability, respectively. In the case of PS-NP, no detectable signal could be obtained for the concentration of 100 µg/mL anymore. The results gained from the comet assay revealed no increase in tail intensity for both MWCNTs (Figure 5b). Increased tail intensity values were observed for the highest concentration of Au-NP I, ZnO-NP and PS-NP. For Au-NP I, only a moderate increase of 35% tail intensity was observed, while ZnO-NP- and PS-NP-treatment reached values of 64 and 86%, respectively. As cell viability was barely or not at all detectable for ZnO-NP and PS-NP after 24 h of incubation, respectively, these high tail intensities in the comet assay were caused by the massive fragmentation of DNA during the process of cell death. This also became apparent upon examination of the microscopic appearance of the comets, which were highly damaged and only detectable with the Comet IV software by additional manual adjustments. Hence, the number of detectable comets used for the evaluation was below the number of comets selected for the analysis of all other samples (100 comets per sample). Results of the FADU assay uncovered a dose-dependent decrease in intact DNA after 24 h of incubation with ZnO-NP and PS-NP (Figure 5c). 

**Figure 6:**
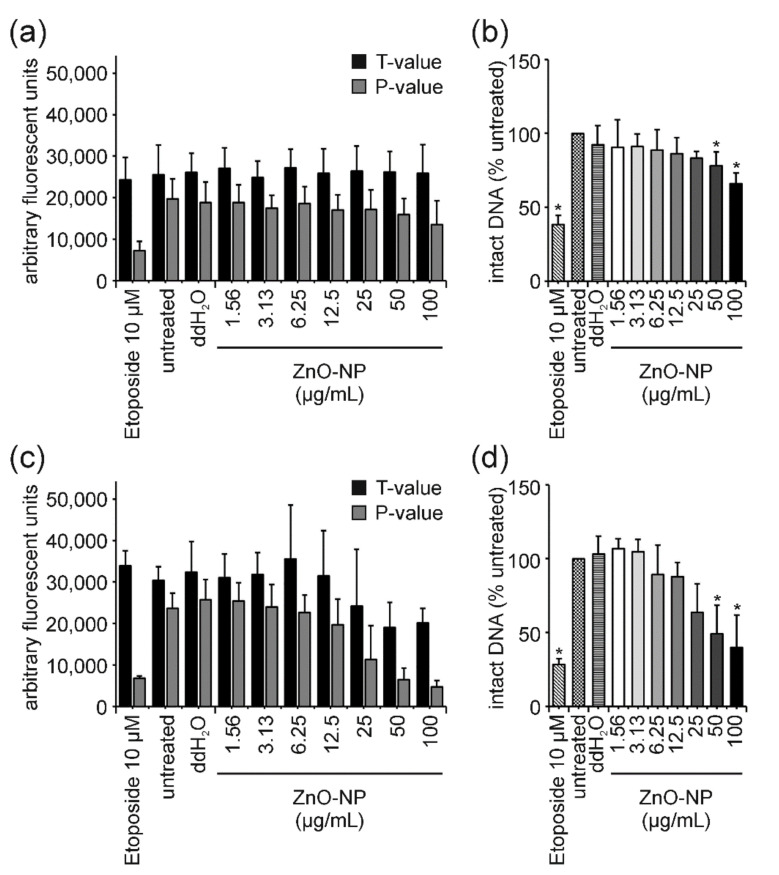
**Influence of ZnO-NPs on T- and *p*-values in the FADU assay and corresponding interference correction**. Following 3 h (**a**,**b**) and 24 h (**c**,**d**) of exposure of Jurkat E6-I cells to ZnO-NP, the FADU assay was performed. After 3 h of incubation, only *p*-values decreased dose-dependently (**a**), while after 24 h, T- and *p*-values decreased with increasing ZnO-NP concentrations (**c**). Results following interference correction for the 3-h (**b**) and 24-h (**d**) time point are shown. Data shown represent the mean of at least three independent experiments and the corresponding standard deviation. (* *p* ≤ 0.05).

**Figure 7:**
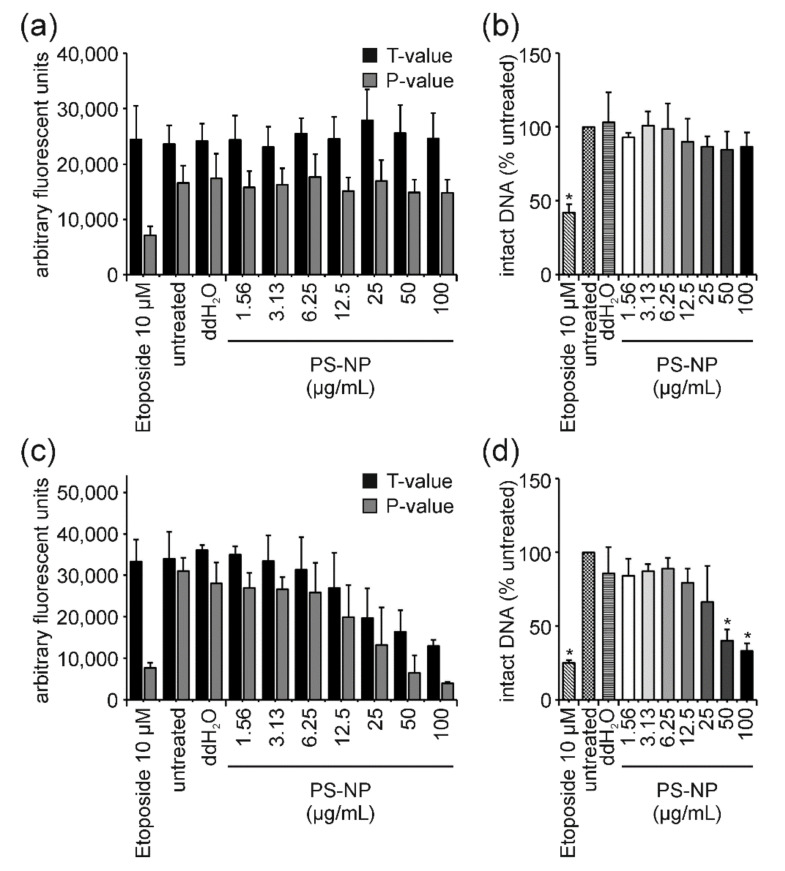
**Influence of PS-NPs on T- and *p*-values in the FADU assay and corresponding interference correction**. Following 3 h (**a**,**b**) and 24 h (**c**,**d**) of exposure of Jurkat E6-I cells to PS-NP, the FADU assay was performed. After 3 h of incubation, only *p*-values decreased dose-dependently (**a**), while after 24 h, T- and *p*-values decreased with increasing PS-NP concentrations (**c**). Results following interference correction for the 3-h (**b**) and 24-h (**d**) time point are shown. Data shown represent the mean of at least three independent experiments and the corresponding standard deviation. (* *p* ≤ 0.05).

For both ENMs, the examination of T-values revealed a strong dose-dependent decrease, similar to what has been described for GO previously. However, in the case of ZnO-NP and PS-NP, this dose-dependent decrease in T-values was not due to interference but caused by cell death and associated DNA fragmentation (Figure 6c,d and Figure 7c,d). This conclusion is supported by two facts: (i) After 3 h of ZnO-NP- and PS-NP-treatment T-values did not decline, even though the same amount of ENMs was present during the reaction and measurement, therefore, confirming the absence of ENM-induced interferences on fluorescence (Figure 6a,b and Figure 7a,b); (ii) Only a few cells were present on the comet assay slides, indicating significant cell loss upon ZnO-NP- and PS-NP-treatment. For cells treated with Au-NP I and MWNT C no significant effects could be observed in the FADU assay (Figure 5c).

It has previously been shown that the toxicity of many metal-based ENMs, including ZnO, TiO_2_, Ag and CeO_2,_ is at least partially caused by their specific properties related to their small size and high surface reactivity. However, in some cases, toxicity may be triggered or further enhanced by the release of free metal ions [40]. The identical batch of ZnO-NP has previously been thoroughly investigated in Jurkat A3 cells and it was shown that the observed cytotoxicity was fundamentally dependent on the fast extracellular dissolution of ZnO-NP and resulting high concentrations of Zn^2+^ ions, which were taken up by the cells [41]. To understand the role of free zinc with the observed ZnO-NP cytotoxicity and genotoxicity in Jurkat E6-I cells, the effect of equimolar concentrations of ZnCl_2_ was analyzed with the same set of assays.

Exposure of Jurkat E6-I cells for 3 h to ZnCl_2_ resulted in a likewise dose-dependent decrease in cell viability in comparison to equimolar concentrations of ZnO-NP-treated cells (Figure 3a and Supplementary Figure S2a). After 24 h of incubation, ZnCl_2_ reduced cell viability at the corresponding concentration of 12.5 µg/mL ZnO-NP slightly more (compare with Figure 5a and Supplementary Figure S2b). However, overall, very similar results were obtained, which suggest that the observed ZnO-NP-induced cytotoxicity is, also in these cells, mainly caused by the release of Zn^2+^. Equivalent results were also obtained in the comet assay where the highest concentration of ZnCl_2_ induced tail intensity values of 56 and 74% after 3 and 24 h of treatment, respectively (Supplementary Figures S2c and S2d), which is comparable to the data acquired for 100 µg/mL ZnO-NP (Figure 3b). Likewise, in the FADU assay, ZnCl_2_- and ZnO-NP-treatment for 24 h dose-dependently reduced the level of intact DNA to the same extent (Figure 3c and Supplementary Figure S2f). Furthermore, T-value controls for both treatment conditions and time points behaved in a comparable manner (Figure 6 and Supplementary Figure S3). The only difference between ZnCl_2_- and ZnO-NP-treatment became apparent after 3 h of treatment in the FADU assay. While ZnO-NPs induced a true genotoxic response at 100 µg/mL (Figure 3c and Figure 6b), no reduction in intact DNA could be observed for equivalent amounts of ZnCl_2_ (Supplementary Figure S2e) indicating an as yet uncharacterized additional nano-effect at early time points in the FADU assay.

## 4. Discussion

At present, there is only limited comparability and consequently a great uncertainty regarding the genotoxic potential of various ENMs. Despite the growing literature on ENM genotoxicity in human cell lines, the published results are quite often controversial due to the different cell types, different ENM types and different readout systems used. In general, including regulatory purposes, the evaluation and interpretation of in vitro data should rely on standardized and solid experimental results. To avoid experimental, assay specific artifacts, it is mandatory to assess each endpoint by two independent, complementary methods that rely on different measurement principles. Such an approach minimizes the probability of systematic or assay-intrinsic errors, which is of particular importance concerning the detection and avoidance of ENM-induced interferences [37]. Not all modes of genotoxic action can be addressed with this study; therefore, we focused, in particular, on a reliable and reproducible methodology for the detection of DNA strand breaks. We investigated the DNA damaging potential of ten different ENMs with distinct properties, thus, aiming for a first preliminary structure–activity relationship analysis. The two independent methods used were the alkaline comet assay as the most frequently used method to assess DNA strand breaks and the novel FADU technology for efficient and semi-automated DNA damage detection [42]. Even though both assays measure DNA strand breaks as the endpoint, only the alkaline comet assay is able to detect additional ALS [35]. Therefore, a careful comparison of results obtained with the comet and the FADU technique is of great importance; in particular, to gain first insights into the mode of action of a certain genotoxicant. In the following paragraphs, the genotoxicity results obtained with the two methods are discussed for each type of ENM in relation to cell viability data. 

### 4.1. TiO_2_-NP: The “Easy One” Neither Induces Cyto- Nor Genotoxicity and Does Not Interfere in the FADU Assay

Cell viability of Jurkat E6-I cells was not affected by treatment with TiO_2_-NP after 3 and 24 h (Figure 1a and Figure 4a). The data on ENM genotoxicity obtained by the alkaline comet assay and the FADU assay are in good agreement showing no genotoxic potential in both assays (Figure 1b,c and Figure 4b,c). Furthermore, no interference with the fluorescent readout in the FADU assay could be observed. While these results are highly consistent in themselves, contradictory findings concerning TiO_2_-NP (geno)toxicity have been published. For instance, Gosh and coworkers and Khan and coworkers [43,44] reported the genotoxic effects of two different types of TiO_2_-NP in human lymphocytes analyzed by comet assay. In contrast, a study by Hackenberg et al., 2011, reported no genotoxicity for these cells after treatment with a distinct type of TiO_2_-NP. Likewise, contradictory results were reported for various cell lines. In human nasal mucosa cells and TK6 cells, no TiO_2_-NP induced genotoxicity could be determined by comet assay, whereas Jugan and coworkers [45] could demonstrate that several different TiO_2_-NPs elicited genotoxic effects on A549 cells [45,46,47]. Since distinct types of TiO_2_-NPs have been used in these studies, a direct comparison is not feasible and a generalized conclusion for all TiO_2_-NP subtypes regarding their genotoxicity and cytotoxicity is still not possible today. Here, we could demonstrate the absence of genotoxicity by means of two independent methods. Additionally, no genotoxic effects following TiO_2_-NP-treatment could be detected in A549 cells (data not shown). This is in agreement with results from Hackenberg and colleagues (2011), who applied the same type of TiO_2_-NP. This allows for the conclusion that this particular type of TiO_2_-NP induces neither cytotoxicity nor genotoxicity in different cell lines in vitro. Further SAR studies are still urgently needed to reduce the need for time-consuming case-by-case evaluations. The assay combination presented here could serve as an ideal platform suitable for diverse cellular models.

### 4.2. GO: The “Interfering One” Does Not Induce DNA Damage but Showcases Interference Reactions in the FADU Assay

Cell viability of Jurkat E6-I cells remained unaffected at all GO concentrations analyzed and up to 24 h of treatment (Figure 1a and Figure 4a). Likewise, tail intensity values measured in the comet assay did not exceed those of the untreated control cells (Figure 1b and Figure 4b). Surprisingly, a dose-dependent decrease in the percentage of intact DNA was observed for increasing concentrations (≥12.5 µg/mL) of GO when analyzed in the FADU assay. This effect was observed for both time points in a very similar manner (Figure 1c and Supplementary Figure S1). GO has previously been shown to interfere with different fluorescence-based assays due to nanoscale-surface energy transfer effects from fluorophores to GO [48,49,50,51,52]. Therefore, analogous interference reactions with the SybrGreen^®^ fluorophore used in the FADU assay could explain the discrepancy between FADU and comet assay results.

In the FADU assay, T-values represented the total amount of DNA in each sample. The pH in these control samples was kept constant to avoid alkaline unwinding even at sites of DNA breakage. Therefore, T-values were directly proportional to the number of cells per sample and were expected to remain constant upon treatment with true genotoxicants, which induce DNA damage but do not affect cell viability, i.e., the number of viable cells. It shall be stressed again that concomitant cell death analyses were indispensable to assure unchanged numbers of viable cells upon treatment. 

GO-treatment reduced T-values in a dose-dependent manner (Figure 2a and Supplementary Figure S1a) and “in parallel” to *p*-value reduction. Together with unchanged cell viability (Figure 1a), this indicated that interference reactions indeed took place in the FADU assay and could be quantified using the T-value controls. Mathematical interference correction, as described in Section 2, eradicated the ostensible genotoxic effect on Jurkat E6-I cells (Figure 2b and Supplementary Figure S1b). Thus, we could establish a straightforward and easy-to-use experimental setup to quantify and mathematically correct for ENM-induced interferences in the FADU assay making it a suitable tool to screen for ENM-induced DNA damage. Moreover, we could clearly demonstrate that GO is neither cytotoxic nor induces DNA strand breaks in Jurkat E6-I cells under the experimental conditions chosen.

### 4.3. SiO_2_-NP of Different Porosities: The “Unclear Ones” Neither Induce Cytotoxicity Nor DNA Damage but Lead to Unclear Results in the FADU Assay

Different types of SiO_2_-NPs have previously been shown to induce negative and positive results in different genotoxicity assays, including the in vitro micronucleus, the comet and the mutation assay with various cell systems [53,54,55,56,57,58,59]. The panel of ENMs analyzed in this study comprised three different SiO_2_-NPs of distinct porosity. As demonstrated by the results of the MTT assay, none of the SiO_2-_NPs reduced cell viability after 3 and 24 h of incubation (Figure 1a and Figure 4a). Likewise, no increase in tail intensity could be observed in the comet assay at either time point (Figure 1b and Figure 4b). The absence of DNA damage was further confirmed after 3 h of treatment by results of the FADU assay (Figure 1c). However, the mesoporous silica sample, MS-SiO_2_-160, led to a minor reduction in intact DNA after 24 h of incubation with 25 and 50 µg/mL. No dose-dependency could be observed, and the level of intact DNA increased again at 100 µg/mL, making a cellular response rather unlikely and hinting toward an interference phenomenon. Mesoporous ENMs are frequently used as carrier systems for different molecules, drugs or dyes. Therefore, it is possible that with an increasing concentration of ENMs an increasing proportion of SybrGreen^®^ was trapped within the pores of the particles [60,61]. This effect might be covered at the highest applied concentration of mesoporous SiO_2_-NPs due to their intrinsic scattering properties, which could prevail at this high density of ENMs [62]. However, a comparable reaction would also be expected after 3 h of incubation and for T-values—both of which was not the case. T-values did not change upon MS-SiO_2_-160-treatment at both time points analyzed (Supplementary Figure S4), indicating no classical interference with the fluorescence signal. Nevertheless, scattering properties of the ENMs could change over time in a protein-containing cell culture medium due to the formation of a protein corona and particle agglomeration leading to an as yet unidentified interference reaction. Taking into account the rather high variability of the FADU assay results, the comparably small effect of MS-SiO_2_-NP on intact DNA and the absence of DNA strand breaks in the (more sensitive) comet assay, the validity of the FADU results for this particular type of ENM is questionable. Furthermore, the absence of DNA strand breaks, as demonstrated in the comet assay, is in accordance with the vast majority of published genotoxicity studies on various SiO_2_-NPs [59,63,64]. Thus, it is evident that more in-depth studies are required to elucidate the mode of action of different silica particles in the FADU assay to eventually make it a reliable screening tool for this particular type of ENM.

### 4.4. PS-NP: The “Purely Cytotoxic” One Induces High Levels of Cytotoxicity thereby Generating False-Positive DNA Damage Results and Could Serve as a Benchmark Material

Exposure of Jurkat E6-I cells to PS-NP for 3 h resulted in a slight dose-dependent decrease in cell viability at the two highest concentrations (Figure 3a). After 3 h, neither in the comet nor FADU assay, was an induction of genotoxicity observed at any concentrations analyzed (Figure 3b,c). This indicates, that in these cells, a reduction in cell viability down to 66%, as measured by the MTT assay, does not trigger considerable DNA fragmentation. DNA fragmentation due to cell death is known as a potential cause of false-positive genotoxicity results in different in vitro assays (e.g., [65,66]). We can, therefore, conclude that PS-NPs induce cytotoxicity but do not induce DNA strand breaks at early time points. Following 24 h of PS-NP incubation, cell viability was strongly reduced in a dose-dependent manner (Figure 5a). For the highest concentration, almost no viable cells could be detected anymore. This was also reflected in a reduced number of nuclei available for comet assay analysis. The remaining nuclei showed a strong increase in tail intensity which was not observed for sublethal concentrations (Figure 5b). Consistently, a significant dose-dependent reduction in the percentage of intact DNA was obtained in the FADU assay (Figure 5c). These observations demonstrate that at least high concentrations of PS-NPs induce pure cytotoxicity and that the observed DNA damage can be attributed to DNA fragmentation secondary to cell death. An important question is how to interpret the dose-dependent effects observed at lower concentrations in the FADU assay. Looking at T and *p* values after 24 h of PS-NP-treatment (Figure 7c) shows an equivalent dose-dependent reduction in T-value signals indicating either a nanomaterial induced interference (as described for GO) or a loss in cell number. Since T values did not change in the 3 h samples, classical nanomaterial interference can be excluded. Hence, the reduction in intact DNA is not based on genotoxic effects but merely on the destruction of DNA integrity due to cell death. 

It is still unknown at which level of cytotoxicity false-positive effects appear in genotoxicity assays and how these are affected by the mode of cell death [67]. Different suggestions for adequate substance concentrations to be used in genotoxicity assessments exist in the literature and cell viability ranges from 70 to 90% [68]. Furthermore, optimal concentration ranges can be affected by the choice of assay used for determination of cell viability, as a reduction in metabolic activity (e.g., by MTT assay) might occur at lower concentrations in comparison to, for example, cytolysis.

Thus, PS-NPs could serve as a benchmark material to determine the level of cytotoxicity at which false-positive genotoxicity results can be expected in the cell type under investigation and the corresponding cytotoxicity assay used.

### 4.5. ZnO-NP: True Genotoxicity vs. Pure Cytotoxicity

A plethora of in vitro studies on ZnO-NP toxicity has previously shown significant effects on the viability of cancer cell lines of the immune system, lung, kidney, skin, and the gut, as well as in primary cells such as neural stem cells, T-lymphocytes or fibroblasts [41,69]. ROS formation and a severe oxidative stress response [70,71,72,73,74,75,76,77] as well as DNA damage in various cell types [73,78,79] have been reported. Furthermore, studies described the dissolution of ZnO-NP and associated Zn^2+^ ion toxicity and/or genotoxicity [41,80,81].

In this study, following exposure to ZnO-NP, the cell viability of Jurkat E6-I cells decreased in a dose-dependent manner after 3 h to approximately 70% for the highest concentration (Figure 3a). For the same exposure time, results of the comet assay revealed a slight trend in DNA damage induction at sublethal concentrations (3.13 and 6.25 µg/mL) and a significant induction of 45% tail intensity at the highest concentration of 100 µg/mL (Figure 3b). Consistently, the amount of intact DNA as measured in the FADU assay, was significantly reduced at 100 µg/mL. In comparison to PS-NP results, this increase in DNA damage upon ZnO-NP-treatment in both assays was rather unexpected. While PS-NP and ZnO-NP show very similar cytotoxicity profiles, no induction of DNA damage was observed for PS-NP after 3 h of treatment. This comparison indicates that even though cell viability (i.e., metabolic activity) is already affected, DNA damage is still induced simultaneously and independently of cell death and can, thus, be interpreted as a real genotoxic effect at early time points. This conclusion is further supported by the analysis of T- and *p*-values in the FADU assay (Figure 6). After 3 h of incubation, T-values were not affected by ZnO-NP treatment—not even at the highest concentrations analyzed. Therefore, the reduction in *p*-values can be considered as a real genotoxic event.

In contrast, after 24 h of ZnO-NP-treatment, a massive reduction in cell viability was observed reaching values of only 2% for the highest concentration (Figure 5a). This led to a strong increase in tail intensity in the comet assay (Figure 5b) and a significant reduction in the percentage of intact DNA in the FADU assay (Figure 5c). All dose–response curves are highly similar to those observed for 24-h PS-NP-treatment. Accordingly, the number of analyzable nuclei at 100 µg/mL of ZnO-NP was markedly reduced and the respective T-values in the FADU assay declined with increasing ZnO-NP concentrations. Nanomaterial-induced interferences could be excluded due to the constant 3-h T-values. Thus, we can conclude that after prolonged exposure to ZnO-NP the observed DNA damage is due to DNA fragmentation in the process of cell death. 

As described earlier, in vitro cytotoxicity of ZnO-NPs can be caused by the high solubility and release of free Zn^2+^, which can lead to the disruption of cellular Zn homeostasis associated with the loss of cell viability, oxidative stress and mitochondrial dysfunction [69,82,83]. The same batch of ZnO-NP induced a caspase-independent alternative apoptosis pathway independent of ROS formation in the Jurkat subclone A3 [41]. This was a consequence of the extracellular release of high amounts of Zn^2+^ followed by rapid cellular uptake. In this study, it could be shown that ZnCl_2_-treatment induced the same dose–response curve in the MTT assay as ZnO-NPs, suggesting that Zn^2+^ ions are responsible for ZnO-NP-induced cell death. Similarly, ZnO-NP- and ZnCl_2_-treatment induced comparable effects on DNA damage in the comet and FADU assay with one exception: the 3-h real genotoxic effect of ZnO as measured in the FADU assay. This was not observed upon ZnCl_2_-treatment. The most likely explanation is that ZnO-NPs and Zn^2+^ ions induce distinct types of DNA lesions at early time points of treatment. As previously analyzed in great detail [35], ALS can only be detected using the alkaline comet assay. Therefore, our results indicate that ZnO-NPs induce DNA strand breaks and, in addition, ALS. In contrast, ZnCl_2_-treatment results in ALS only. Since these specific lesions cannot be detected by the FADU assay, no reduction in intact DNA could be observed upon 3 h of ZnCl_2_-treatment.

### 4.6. Au-NP: True Genotoxicity vs. Pure Cytotoxicity and a Potential Mechanism of Action

Gold is generally considered an inert and biocompatible material. Consequently, for a long time, Au-NPs were expected to behave similarly and to be non-toxic [84,85]. However, various publications with often contradictory findings on cytotoxicity and genotoxicity of Au-NPs appeared over the past years. For example, Au-NPs with a size of 1–2 nm were reported to induce a high cytotoxicity, while 15 nm-sized Au-NPs were non-toxic in different cell lines [86]. Similarly, 5 nm-sized Au-NPs induced genotoxic effects, whereas 50 nm-sized Au-NPs did not [87]. While such a size-dependency for cytotoxicity as well as for genotoxicity was observed in different studies [88,89], opposing reports have also been published [90,91], thereby explaining the need for a reliable genotoxicity assessment of Au-NPs.

Our results demonstrate that the cell viability of Jurkat E6-I cells was slightly reduced after 3 h of treatment with Au-NP I (Figure 3a). This dose-dependent effect was more pronounced after 24 h of incubation (Figure 5a). Interestingly, the comet assay revealed an increase in tail intensity after 3 h and 24 h, indicating that DNA damage induction by these ENMs (Figure 3b and Figure 5b). However, results of the FADU assay showed no significant reduction in intact DNA at both time points (Figure 3c and Figure 5c). This discrepancy can be explained by differences in the sensitivity of these two methods regarding certain types of DNA lesions. As published by Singh et al., the detection limit of the comet assay is 0.03 Grey, while the FADU assay reaches a detection limit of 0.1 Grey [92]. While X-ray-induced DNA damage is already measured with greater sensitivity in the comet assay, differences for other genotoxic stimuli (causing distinct kinds of lesions) might be even more pronounced. Both methods detect DNA strand breaks as general endpoints but they are based on different measurement principles and do not necessarily detect the same spectra of DNA lesions.

Our previous study [35] demonstrated the importance of pH to also detect ALS. While the same Au-NP I induced very high DNA damage levels in A549 cells in the alkaline comet assay (performed at pH 13.2–13.7), only a weak DNA damage induction was observed in the neutral comet and the FADU assay (performed at pH 12.5–12.9; [20]). These results led to the conclusion that mainly ALS, which can be detected as strand breaks under extremely high pH of 13 and above, are induced by these NPs. The same phenomenon could explain the results observed in Jurkat E6-I. However, another remaining question is whether the assumed DNA-damaging effect is due to genuine genotoxicity and in consistency with previously published data [35] or due to cell death, which was not observed in A549 cells. Comparing the Au-NP cytotoxicity results to the proposed benchmark material PS-NP, the reduction in cell viability after 3 h of treatment was still in a range where no DNA fragmentation would be expected. Since genotoxicity was still detectable in the comet assay, this can be attributed to genuine DNA damage. In addition, Au-NPs did not affect T-values in the FADU assay at any concentrations and time points analyzed. This further supports the conclusion that the observed increase in tail intensity is not primarily due to cell death but rather a true genotoxic event. However, after 24 h of exposure at least the highest concentration (100 µg/mL) of Au-NP I would be expected to lead to DNA fragmentation due to considerable cell death (Figure 5). The lack of a genotoxic effect measured by the FADU assay indicates that the DNA fragmentation did not yet reach a significant level. The increase in tail intensity as measured by the comet assay could, thus, be due to ALS, as previously shown [35] and discussed above.

### 4.7. MWNTs: Cytotoxicity without DNA Damage—Is That Possible?

Following 3 h of incubation with MWNT A and C, cell viability was reduced dose-dependently to 32 and 44% for the highest concentration, respectively (Figure 3a). This effect increased after 24 h of incubation down to 30 and 25%, respectively (Figure 5a). At such low levels of cell viability and in relation to the proposed benchmark material (PS-NP), DNA fragmentation was expected to influence the comet as well as FADU assay results. However, in neither was assay DNA damage was observed (Figure 3 and Figure 5). Furthermore, no influence on T-values could be detected (Supplementary Figure S5). On the one hand, this indicates that MWNTs do not interfere with the fluorescence readout of the FADU assay. On the other hand, a massive loss of viable cells can also be excluded. This would suggest that MWNTs influence metabolic activity to a greater extent without impacting on actual cell death as, for example, PS-NP-treatment. Furthermore, MWNTs are known to interfere with the MTT assay and even though interference controls have been run in this study (data not shown), an overestimation of the cytotoxic potential is still possible [93]. Further analysis utilizing additional cell viability assays and a more detailed analysis on genotoxicity are needed to elucidate the remaining ambiguities. Nevertheless, the data presented here allow for the conclusion that, at sublethal concentrations, none of the investigated MWNTs induced DNA strand breaks in Jurkat E6-I cells.

## 5. Conclusions

With the set of ENM provided and corresponding results in combination with two complementary, yet independent, genotoxicity assays and the implemented controls, we believe that genotoxicity assessment can be improved and brought to the next level of reliability. False-positive genotoxicity results can be avoided, and true genotoxicity can be detected with a high level of confidence.

Even though a classical SAR could not be deduced from the data set provided, we can still conclude that porosity in the case of silica particles neither influenced assay results (interference) nor cytotoxicity and DNA damage in Jurkat E6-I cells. Likewise, shape (2D vs. spheroidal vs. tubes) does not seem to be the decisive factor in terms of cytotoxicity and DNA damage under the experimental conditions chosen. We can further conclude that TiO_2_-NPs, GO, all three types of SiO_2_-NPs as well as MWNT A and C do not induce DNA strand breaks in Jurkat E6-I cells. Using PS-NPs as a purely cytotoxic benchmark material allowed us to identify the true genotoxic potential for ZnO-NP at a short (i.e., 3 h) exposure time as well as for Au-NP I at both exposure times analyzed. Further relating the ZnO-NP results to ZnCl_2_ data and previously published findings on Au-NP I revealed a potential nano-specific genotoxic effect for ZnO-NP that was not caused by the release of Zn^2+^ ions. Based on GO results, we established an easy-to-use quantitative interference control for the FADU assay making it a promising screening tool for ENM genotoxicity. We believe that the approach described here will be applicable for any cell type of interest, given that a suitable cell-type-specific, purely cytotoxic benchmark material is available. 

## Figures and Tables

**Table 1:** ENM suspension preparation.

	Delivered as	Prepared Stock Concentration	Solvent	Ultrasonication ^1^
**PS-NP**	100 mg/mL in ddH_2_O		ddH_2_O	-
**TiO_2_-NP**	powder	1 mg/mL	ddH_2_O	10 min
**ZnO-NP**	powder	1 mg/mL	ddH_2_O	10 min
**Au-NP I**	4.7 mg/mL in ddH_2_O		ddH_2_O	-
**MWNT A**	powder	0.5 mg/mL	Pluronic F-127	10 min
**MWNT C**	powder	0.5 mg/mL	Pluronic F-127	10 min
**GO**	powder	1 mg/mL	ddH_2_O	2 min
**SiO_2_-160**	11.2 mg/mL in ddH_2_O		ddH_2_O	-
**MS-SiO_2_-140**	4.7 mg/mL in ddH_2_O		ddH_2_O	-
**MSHT-SiO_2_-300**	18.6 mg/mL in ddH_2_O		ddH_2_O	-

^1^ Sonication using an ultrasound bath.

**Table 2:** Characteristics of cytotoxic ENM.

Description	Au-NP	MWNT A	MWNT C	ZnO-NP	PS-NP
**Source**	collaboration partners of the CCMX NanoScreen consortium ^a^	Bayer Technologies Service, Baytubes, Leverkusen, Germany	Cheap Tubes Inc., Grafton, Vermon, USA	IBUtec, Weimar, Germany	Bangs Laboratories, Inc., Fishers, IN, USA
**Delivered as**	suspension (4.7 mg Au/mL in ddH_2_O)	powder	powder	powder	suspension (100 mg/mL in ddH_2_O)
**Manufacturing process**	see Bohmer et al., 2018			pulsation reactor technique	
**Size/Size distribution (diameter)**	TEM: 3.1 ± 1.3 nm DLS ^b^: 147 nm	inner diameter: 1–9 nm outer diameter: 4–24 nm	inner diameter: 2–13 nm outer diameter: 6–34 nm	TEM: 15.5 ± 3.9 nm	57 nm ^c^ SEM: 51 ± 9 nm DLS ^b^: 56 nm
**Lateral dimensions**		1–5 µm	1–16 µm		
**Surface area**				60 ± 5 m^2^/g ^c^	99 m^2^/g ^c^
**Density**	19.3 g/cm^3 d^				1.05 g/cm^3 c^
**Zeta potential ^e^**	24.5 mV	−5 mV in Pluronic F-127	−15 mV in Pluronic F-127	−24.3 mV	48.8 mV
**Surface modification**	[AL]_21_[α-gal]_23_				NH_2_ (amine)
**Publication on characterization details**	Bohmer et al., 2018 Rademacher et al., 2013 patent ^a^	Thurnherr et al., 2009	Thurnherr et al., 2009	Buerki-Turnherr et al., 2013	Elliott et al., 2017

^a^ for details see patent US 8,568,781 B2, 2013. ^b^ DLS values are given as Z-average from measurements in ddH_2_O. ^c^ Manufacturer’s information. ^d^ Density of Au, ratio of NP core to ligands unknown. ^e^ If not otherwise specified zeta potential was measured in water. abbreviations: DLS: dynamic light scattering; MWNT: multi-walled carbon nanotubes; NP: nanoparticle; SEM: scanning electron microscopy; TEM: transmission electron microscopy.

**Table 3:** Characteristics of cytotoxic ENM.

Description	SiO_2_-160	MS-SiO_2_-160	MSHT-SiO_2_-300	TiO_2_-NP	GO
**Source**	collaboration partners of the CCMX NanoScreen consortium ^a^	collaboration partners of the CCMX NanoScreen consortium ^a^	collaboration partners of the CCMX NanoScreen consortium ^a^	Sigma-Aldrich	Cheap Tubes, Inc.
**Delivered as**	suspension (11.2 mg/mL in ddH_2_O)	suspension (4.7 mg/mL in ddH_2_O)	suspension (18.6 mg/mL in ddH_2_O)	powder	powder
**Manufacturing process**	Stöber synthesis	CTAB-method	CTAB-method with additional hydrothermal treatment		modified Hummers method
**Size/Size distribution (diameter)**	TEM: 161 ± 15 nm DLS ^b^: 204 ± 2 nm	TEM: 128 nm DLS ^b^: 209 nm	TEM: 288 nm DLS ^b^: 270 nm	<25 nm ^c^ DLS ^b^: 279 ± 51 nm	thickness: 0.7–1.2 nm ^d^
**Lateral dimensions**					SEM: 1–40 µm AFM: 300–800 nm
**Surface area**	23 m^2^/g ^e^	1092 m^2^/g ^e^	462 m^2^/g ^e^	200–220 m^2^/g ^c^	
**Density**				3.9 g/cm^3 c^	
**Zeta potential ^f^**	−49 ± 3 mV	−35.2 mV	−47.7 mV	−36.1 ± 1 mV	−39.4 ± 1.3 mV
**Publication on characterization details**	Bohmer et al., 2018	unpublished	unpublished	unpublished	Kucki et al., 2016

^a^ Powder Technology Laboratory, EPFL, Lausanne, Switzerland. ^b^ DLS values are given as Z-average from measurements in ddH_2_O. ^c^ Manufacturer’s information. ^d^ Corresponds to few- or even single-layer graphene. ^e^ Assessed by N2-BET. ^f^ If not otherwise specified zeta potential was measured in water.

## Data Availability

The data presented in this study are available in the Supplementary Materials: Supplementary_RawData_MTT.xlsx; Supplementary_RawData_FADU.xlsx; Supplementary_RawData_Comet.xlsx.

## Supplementary Materials

The following are available online at www.mdpi.com/article/10.3390/nano12020220/s1, Figure S1: GO-induced interference and interference correction in the FADU assay after 24 h of incubation in Jurkat E6-I cells, Figure S2: Influence of ZnCl_2_ on Jurkat E6-I cell viability and DNA damage induction after 3 h and 24 h of incubation, Figure S3: Influence of ZnCl_2_ on T- and *p*-values in the FADU assay and corresponding interference correction, Figure S4: No influence of MS-SiO_2_-160 on T-values in the FADU assay, Figure S5: No influence of MWNT A and MWNT C on T-values in the FADU assay.
