# Supplementary material for: Assessing Genotoxicity of Ten Different Engineered Nanomaterials by the Novel Semi-Automated FADU Assay and the Alkaline Comet Assay
Source: Nanomaterials (Basel). 2022 Jan 10;12(2):220. doi: 10.3390/nano12020220 (PMC8781421; doi:10.3390/nano12020220)
Supplement: Supplementary file 1 [file nanomaterials-12-00220-s001.zip › nanomaterials-1460998-supplementary.pdf]

nanomaterials

Supplementary Material

Assessing Genotoxicity of Ten Different Engineered Nanomaterials by the Novel Semi-Automated FADU Assay and the Alkaline Comet Assay

Sarah May [1,2], Cordula Hirsch [1], Alexandra Rippl [1], Alexander Bürkle [2] and Peter Wick [1,*]

1 Empa, Swiss Federal Laboratories for Materials Science and Technology, Particles–Biology Interactions, Lerchenfeldstrasse 5, 9014 St. Gallen, Switzerland; sf.may@hotmail.de (S.M.); cordula.hirsch@empa.ch (C.H.); alexandra.rippl@empa.ch (A.R.)
2 Molecular Toxicology Group, University of Konstanz, Universitätsstrasse 10, 78464 Konstanz, Germany; alexander.buerkle@uni-konstanz.de
* Correspondence: peter.wick@empa.ch

Figure S1:

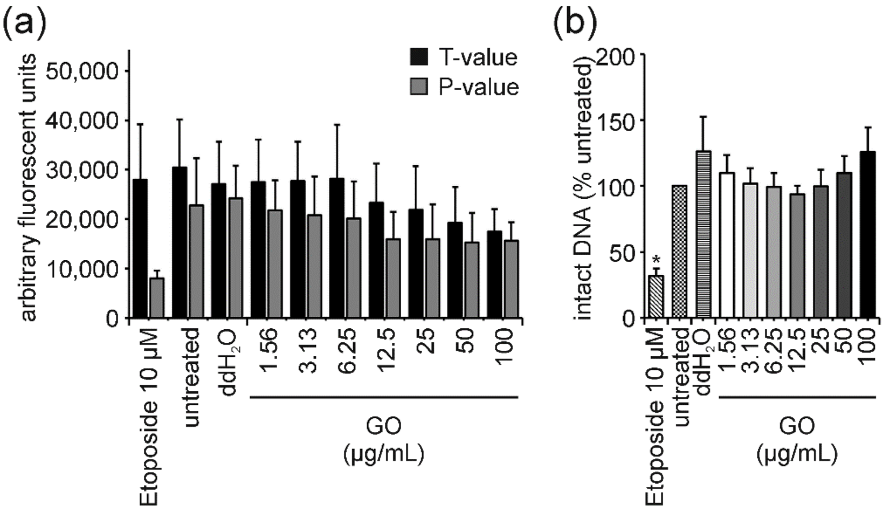

Figure S1. GO-induced interference and interference correction in the FADU assay after 24 h of incubation in Jurkat E6-I cells. Following 24 h exposure of Jurkat E6-I cells to GO the FADU assay was performed and revealed a dose-dependent decrease in fluorescence values of T- and P-values (a). After correction of the observed interference obtained for GO treatment no reduction in percentage intact DNA can be observed for any concentration of GO (b). Only 10 µM etoposide for 30 min, which served as positive controls induced genotoxic effects. Data shown represent the mean of four independent experiments and the corresponding standard deviation. (*$p \leq 0.05$).

Figure S2:

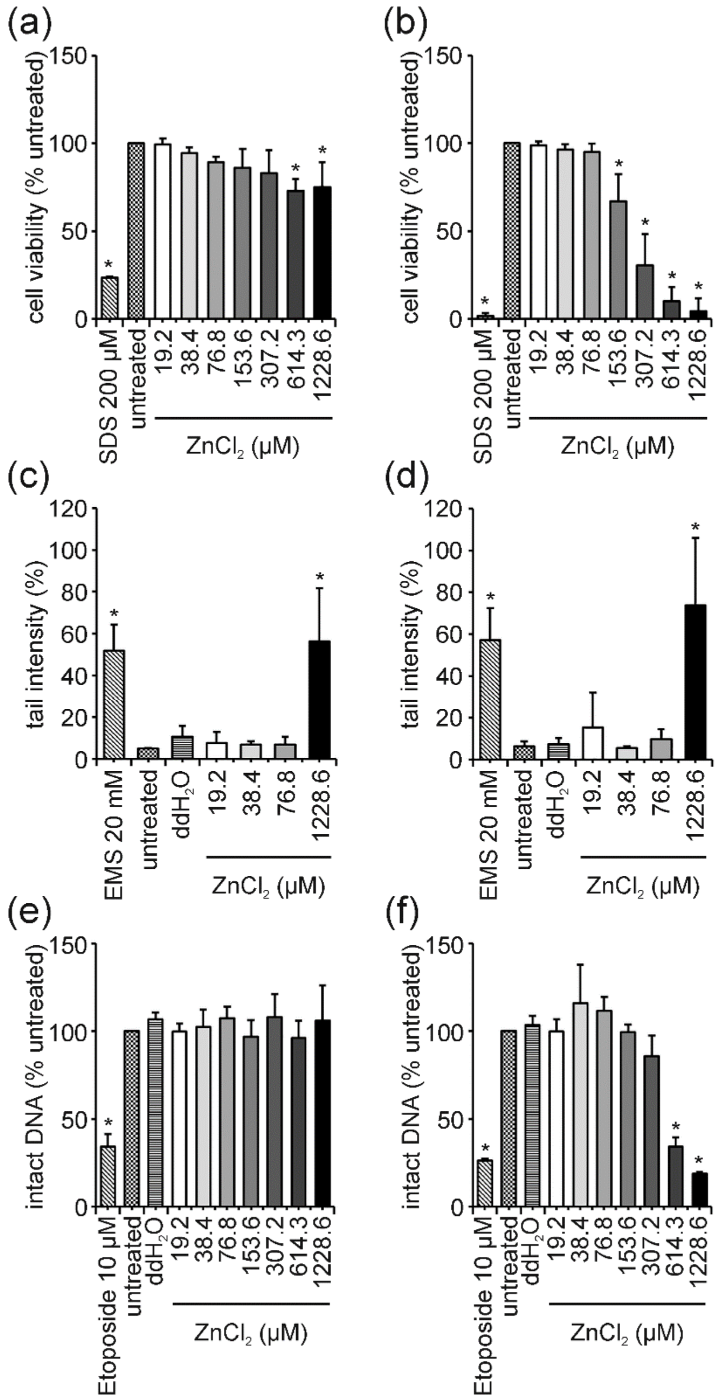

Figure S2. Influence of $ZnCl_2$ on Jurkat E6-I cell viability and DNA damage induction after 3 h and 24 h of incubation. Following incubation of Jurkat E6-I cells with increasing concentrations of $ZnCl_2$, equimolar to the previously applied ZnO-NP concentrations, for 3 h (a) and 24 h (b) cell viability was determined by MTT assay. As a positive control cells were incubated with 200 µM SDS (3 and 24 h). DNA damage expressed as % tail intensity was assessed by alkaline comet assay after 3 h (c) and 24 h (d) of incubation. EMS (30 min) served as the positive control. The FADU assay was performed as second independent for genotoxicity assessment after 3 h (e) and 24 h (f) of incubation. Treatment with etoposide (30 min) served as the positive control. Results represent the mean and corresponding standard deviations from three independent experiments. (*$p \leq 0.05$).

Figure S3:

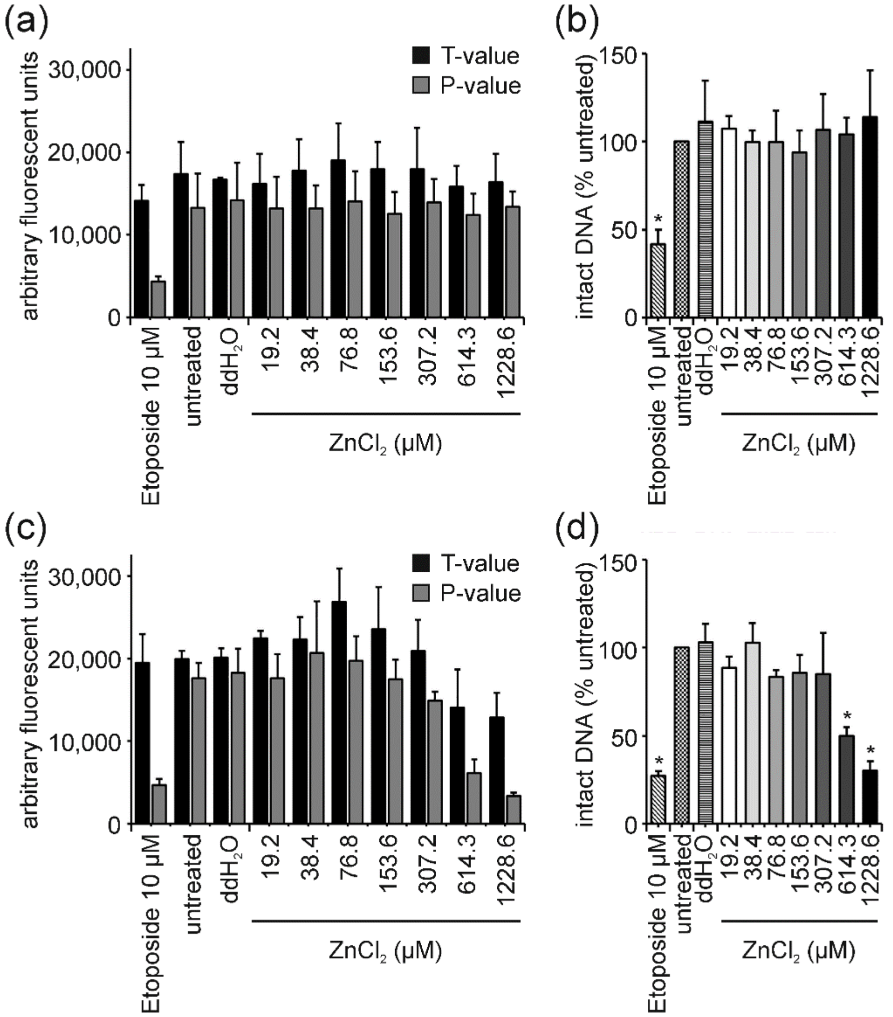

Figure S3. Influence of $ZnCl_2$ on T- and P-values in the FADU assay and corresponding interference correction. Following 3 h (a) and 24 h (c) exposure of Jurkat E6-I cells to $ZnCl_2$ at equimolar concentrations to the previously applied ZnO-NP concentrations, FADU assay was performed. After 3 h of incubation only P-values decrease dose-dependently, while after 24 h both, T- and P-values decrease with increasing $ZnCl_2$ concentrations. Results following interference correction for the 3 h (b) 24 h (d) time point are shown. Data shown represent the mean of three independent experiments and the corresponding standard deviation. (*$p \leq 0.05$).

Figure S4:

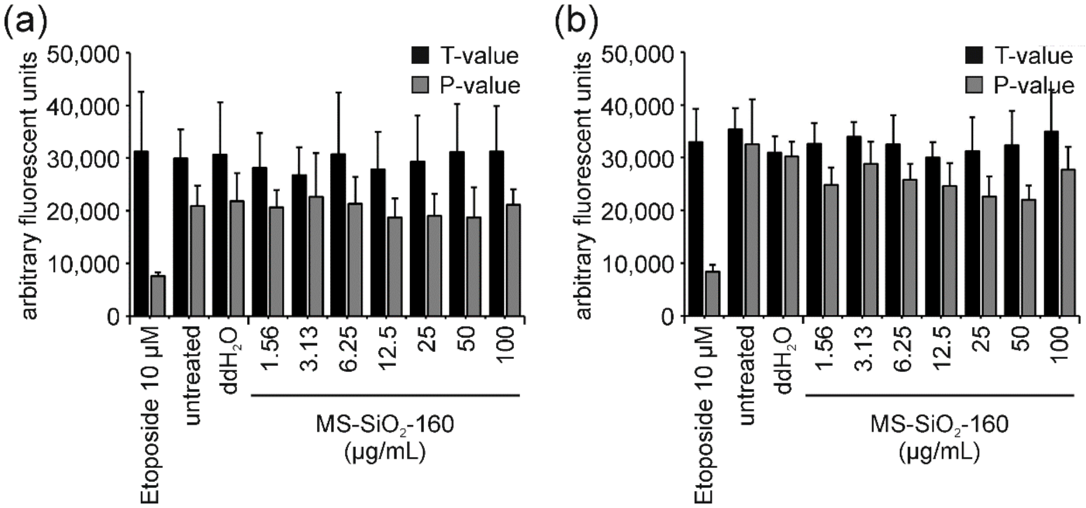

Figure S4. No influence of MS-SiO2-160 on T-values in the FADU assay. Following 3 h (a) and 24 h (c) exposure of Jurkat E6-I cells to MS-SiO2-160 at indicated concentrations FADU assay was performed. Neither after 3 h nor 24 h of incubation a dose-dependent change in T-values upon MS-SiO2-160 treatment could be observed. Data shown represent the mean of four independent experiments and the corresponding standard deviation.

Figure S5:

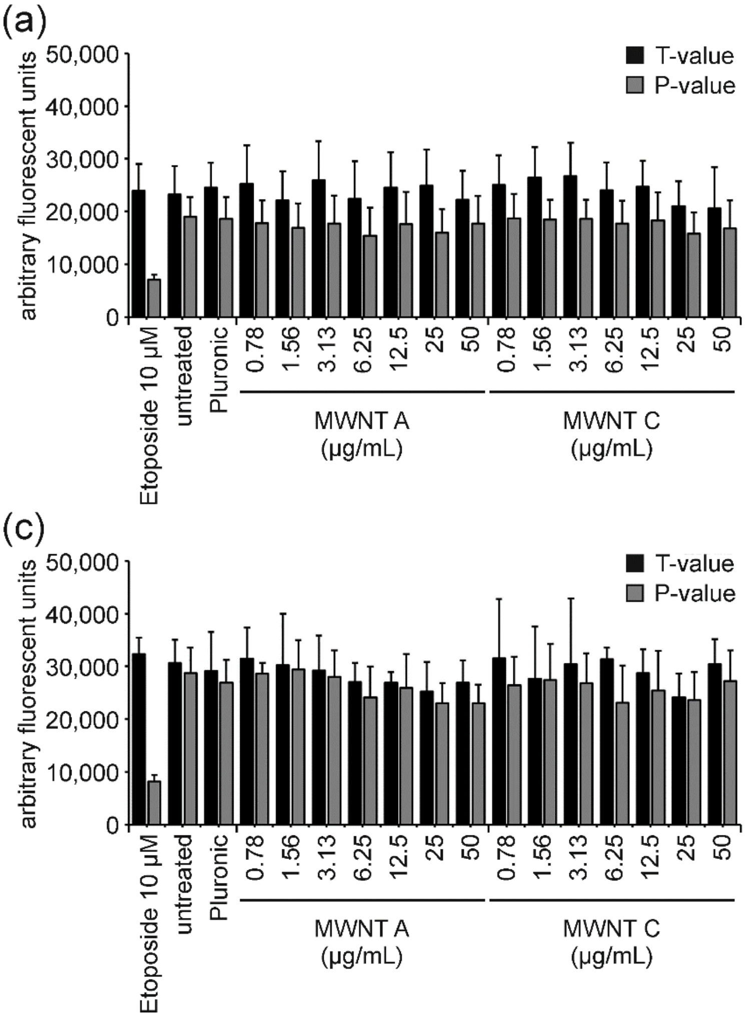

Figure S5. No influence of MWNT A and MWNT C on T-values in the FADU assay. Following 3 h (a) and 24 h (c) exposure of Jurkat E6-I cells to MWNT A and MWNT C at indicated concentrations FADU assay was performed. Neither after 3 h nor 24 h of incubation a dose-dependent change in T-values upon treatment with both MWNT samples could be observed. Data shown represent the mean of three independent experiments and the corresponding standard deviation.
